# A Review of Heavy Metal Contamination of Food Crops in Nigeria

**DOI:** 10.29024/aogh.2314

**Published:** 2018-08-31

**Authors:** Michael Monday Onakpa, Anoka Ayembe Njan, Ogbureke Chidiebere Kalu

**Affiliations:** 1 Department of Pharmacology and Toxicology, Faculty of Veterinary Medicine, University of Abuja, NG; 2 Department of Pharmacology and Therapeutics, College of Medicine, University of Ilorin, Kwara State, NG; 3 Department of Family Medicine, Irrua Specialist Teaching Hospital, NG

## Abstract

Heavy metal contamination of food crops is an issue of global concern that ultimately results in toxicity and diseases in humans and animals through consumption of contaminated soils and food crops. With a population of 182 million people, Nigeria is regarded as the most populous country in Africa. The people suffer environmental pollution from high levels of heavy metal accumulation in the environment and in food crops. Heavy metals have atomic densities higher than 4 g/cm^3^, and these include lead (Pb), cadmium (Cd), zinc (Zn), mercury (Hg), arsenic (As), silver (Ag), chromium (Cr), copper (Cu), iron (Fe), and platinum (Pt). The high level of environmental contamination by these metals is dangerous because their uptake by plants and subsequent accumulation in food crops consumed by humans and animals is deleterious to health. There are many known sources of harmful metals, including the earth, which releases them into food, air, and water, and anthropogenic activities, such as the application of fertilizer in agriculture, the use of pesticides and herbicides, and irrigation. Other sources are automobile emissions, paints, cigarette smoking, industries, and sewage and waste disposal. Evidence shows that vegetables and other food crops consumed in Nigeria are contaminated by heavy metals, and this is associated with adverse health issues, such as cancer, which is currently on the rise in Nigeria. It is therefore vital that communities with high levels of heavy metal pollution avoid eating large quantities of these food items. There is also the need for the monitoring of levels of these injurious elements in food crops.

## Introduction

With a population of 182 million, Nigeria is considered the most populous country in Africa [1]. One of the consequences of the increased human population is environmental pollution, and it is possible to establish relationships between overpopulation, poverty, and urban air pollution [2]. Poverty as an outcome of overpopulation for a country of low gross domestic product results in a majority of its citizens using rickety, old, smoke-belching cars and two-stroke engine motorcycles as a means of public transport, and this has resulted in environmental pollution in most Nigerian cities [3].

A heavy metal is any metallic element that has a relatively high density and is toxic or poisonous even at low concentrations [4]. The term applies to the group of metals and metalloids with an atomic density greater than 4 g/cm^3^, or at least 5 times greater than the density of water [5]. Heavy metals include lead (Pb), cadmium (Cd), zinc (Zn), mercury (Hg), arsenic (As), silver (Ag), chromium (Cr), copper (Cu), iron (Fe), and the platinum (Pt) group.

Heavy metal contamination is a major environmental health challenge and is potentially dangerous because of bioaccumulation through the food chain [6], which arises from rapid industrial growth, advances in the use of agricultural chemicals, and the urbanising activities of man. This has led to the dispersion of heavy metals in the environment, resulting in the impaired health of the population, mainly by the ingestion of food crops contaminated by these harmful elements [7]. Uptake of heavy metals by plants through absorption and subsequent accumulation along the food chain is a potential threat to animal and human health [8,9].

## Sources of Heavy Metals

Heavy metals are natural constituents of the earth’s crust and are persistent environmental contaminants; they are not degradable and enter the body through food, air, and water and bioaccumulate over a period of time [4,10]. They can be released into the environment by natural and anthropogenic sources. Anthropogenic sources of heavy metal contamination include agricultural activities, such as pesticide and herbicide application, contaminated irrigation water, municipal waste used for fertilization [11], and even mineral fertilizer containing traces of heavy metals [12]. Additional anthropogenic sources of heavy metals include direct waste disposal on farmland [13], mining activities, use of lead as antiknock in petrol, traffic emissions, cigarette smoking, metallurgy and smelting, aerosol cans, sewage discharge, and building materials, such as paints [14]. The atmosphere can be loaded with heavy metals through the breakdown of applied waste materials, which gradually release the heavy metals in them. However, lead accumulation on Nigerian soil is a result of long-term cultivation [15], while a significant increase in the concentration of zinc in pasture fields is due to the application of manure [16].

In the savannah region of Nigeria, some heavy metals have been reportedly added to the soil through farmyard and chemical fertilizer application [17]. Heavy metal emissions from other sources, such as worn automobile tires and brake linings, roofs, and food remnants in residences, as well as other domestic byproducts, such as refuse, have also been identified [18,19]. Heavy metals emanating from anthropogenic sources are more dangerous because of their instability and solubility, which leads to a high bioavailability [20].

## Factors Influencing Uptake Of Heavy Metals By Plants

Absorption and accumulation of heavy metals in plant tissues depend upon temperature, moisture, organic matter, pH, and nutrient availability [21]. Heavy metal accumulation also depends on plant species, while the efficiency of plants in absorbing metals is determined by either plant uptake or soil-to-plant transfer factors of the metals [22]. Elevated lead levels in soils for instance may decrease soil productivity, while a very low lead concentration may inhibit some vital plant processes, such as photosynthesis, mitosis, and water absorption, leading to symptoms of toxicity, like dark green leaves, wilting of older leaves, stunted foliage, and brown short roots [23]. Heavy metals are potentially toxic, resulting in chlorosis, weak plant growth, and low yield, and they may even be accompanied by reduced nutrient uptake, disorders in plant metabolism, and a reduced ability to fix molecular nitrogen in leguminous plants [24].

## Contamination Of Soil And Food Crops In Nigeria

Fruits and leafy vegetables are widely used with other foods for culinary purposes, especially for increasing the quality of soups and for their nutritional value [25]. They are part of the daily diet in many households in Nigeria and are a source of vitamins and minerals. They are made chiefly of cellulose, hemi-cellulose, and pectin, which give them their texture and firmness [26]. Consumer perception of better quality vegetables is subjective as they consider dark green and big leaves as characteristics of good quality. However, the external morphology of vegetables cannot guarantee wholesomeness because heavy metals rank high amongst the major contaminants of leafy vegetables [27].

## Northern Nigeria

A study aimed at assessing heavy metal bioaccumulation in spinach, jute mallow, and tomato in farms within Kaduna State revealed that the concentrations of heavy metals in agricultural soil samples were generally higher than the World Health Organization/Food and Agriculture Organization of the United Nations (WHO/FAO) maximum permissive limits for lead and cadmium, but lower for nickel (Ni) and chromium (Cr) [28]. The mean concentrations of heavy metals in vegetables were found to be above the permitted limits for all the heavy metals, except nickel. This calls for urgent attention, especially for lead and cadmium, which are highly toxic and have no known biological use [29].

In another investigation of the variation of metal contents of irrigated vegetable farms in Kano metropolis, it was found that the relative abundance of heavy metals in the farm produce followed the sequence Fe > Zn > Mn > Cu > Ni > Pb > Co > Cr [30]. The relative abundance of heavy metals affects the toxicity of metals to plants and the uptake of these metals by plant roots. Therefore, good quality control for food crops is important to protect consumers from exposure and toxicity [30].

## Central Nigeria

In the Itakpe iron ore mining site of Kogi State, heavy metal concentrations in crops were reportedly higher than those grown on control soil even though the observed concentrations of heavy metals were below the WHO/FAO limit for food [31]. The presence of zinc and copper in plants and the higher level of metals observed in the mining site indicate that agrochemicals and mining contribute to high levels of heavy metals in the environment.

Vegetables constitute the lowest source of essential trace elements for people in developing countries like Nigeria. A study of copper and zinc contents in vegetable samples in this region showed that copper concentrations were 5 mg/kg in peppers, 4 mg/kg in onions, and 5.5 mg/kg in tomatoes, while zinc levels were 13.5 mg/kg in peppers, 16.75 mg/kg in onions, and 15.5 mg/kg in tomatoes [32]. These findings indicate that vegetables from this region could serve as good dietary sources for essential trace metals, and the levels were within the safety margin for human consumption [27].

## Western Nigeria

*Amaranthus* grown along major highways in Lagos State showed a decrease in heavy metal concentrations with an increase in distance from the road. Concentrations in *Amaranthus* cultivated on soils characterized by heavy traffic were higher than those cultivated on reference soil [33], suggesting that *Amaranthus* can concentrate heavy metals in their tissues and that aerial deposition may be a major source of contamination. It is also true that a strong relationship exists between heavy metals in the soil and contamination of farm produce; therefore, the consumption of leafy vegetables and crops produced on contaminated soils can pose a health risk [34].

In another study aimed at assessing the levels of zinc, manganese, cobalt, selenium, copper, molybdenum, chromium, iron, aluminium, lead, and cadmium in different plant parts and the index of bioaccumulation (ratio in plant/soil) in Ekiti State, zinc and iron were mostly concentrated in the plant organs, while manganese was found in very few plants [35]. The indices of bioaccumulation for zinc and iron ranged from medium to intensive in iron but only intensive for zinc [35]. Similarly, the heavy metal concentrations in plant leaves and crops showed high levels of cobalt (0.33mg/kg) and iron (0.32mg/kg) in Roselle leaves, copper (0.71mg/kg) and arsenic (0.37mg/kg) in groundnut, copper (0.48mg/kg) and arsenic (0.28mg/kg) in maize grains, arsenic (0.36mg/kg) and cobalt (0.32mg/kg) in spinach leaves, and copper (0.36mg/kg) and cobalt (0.32mg/kg) in okro [36]. The samples from dumpsites had higher levels of contamination, suggesting a possible mobility of metals from dumpsites to farmlands through leaching and runoffs.

Iron levels reduced by 8.25% in onions and nickel by 45.19% in okro in rainy season samples over those of the dry season, and the mean levels of metals in the okro samples for the dry season were in the order Fe > Cu > Zn > Mn > Ni > Pb > Co > Cr, while those of the rainy season indicated Fe > Cu > Zn > Mn > Ni > Co > Pb > Cr [37]. This trend suggests that okro has a higher retention capacity for essential metals, zinc, manganese, and copper, than for the toxic ones, nickel, lead, cobalt, and chromium [35]. It was observed that the general transfer factors (potentiality of heavy metals to be absorbed by plants) for vegetables are in the order of Cd > Zn > Hg > Pb. These might be due to a higher mobility of cadmium occurring naturally in soil [38] and the low retention of cadmium in the soil than other toxic cations [39]. The associated health risk assessment of heavy metals to consumers shows that lead, zinc, and mercury in the vegetables studied were above the FAO/WHO permissible limits [40]. The health risk values of estimated daily intake of metal (DIM) (the presumed daily exposure to or consumption of a heavy metal) and health risk index (HRI) (the capacity of a toxicant to pose a danger on exposure if it is greater than one for any metal in food crops is an indication that the consumer population faces a health risk) indicate that lead, cadmium, and mercury contamination in the vegetables carried higher health risk indexes [41].

## Eastern Nigeria

During a population health risk assessment on the consumption of heavy-metal-contaminated food crops and fruits in Owerri, Imo State, it was reported that the concentration of lead, cadmium, and nickel exceeded the maximum allowable concentrations for agricultural soil as recommended by the European Union [42]. Levels of lead, cadmium, and nickel in food crops were highest in *Oryza sativa, Glycine max*, and *Pentabacta microfila* respectively. The highest levels of lead, cadmium, and nickel in fruits were detected in *Canarium schweinfurthii, Citrus reticulata*, and *Ananas comosus*, respectively [42]. It is therefore important to note that local foodstuffs commonly consumed in eastern Nigeria may contribute to the burden of heavy metal, and this is of public health importance. This is alluded to by the fact that heavy metals were found in rice samples in Enugu State at levels above the WHO maximum permissible limit. Hazard quotients and total hazard index for lead and cadmium in these studies were greater than 1 [43].

Lead levels in spices such as *Prosopis africana, Xylopia aethiopica, Piper gineense, Monodora myristica*, and *Capsicum frutescens* in Awka, Ebonyi State, were 8 to 30 times higher than the WHO/FAO permissible limit. This implies that spices consumed in this region may add to the burden of lead [44].

## The Niger Delta Region

The Niger Delta, comprised of nine states greatly endowed with abundant natural resources, gives rise to increased industrial activities [45]. Environmental degradation of the oil-rich region has caused wanton destruction and continuous harm to the physical, social, and economic health of its people. Petroleum refineries produce a wide variety of air and water pollutants and hazardous solid wastes [46].

Studies on the concentration of trace metals in crops harvested in some oil exploration sites in Rivers State revealed that the mean concentration of lead was 1.1mg/kg in cocoyam and 9.1mg/kg in okro [47]. This result reflects a higher concentration of heavy metals in crops from the industrialized locations, with green vegetables having the highest uptake than other crops. These findings call for concern, particularly as heavy metals bioaccumulate and pose a serious health risk to man and animals. The concentration of zinc, cadmium, lead, iron, copper, chromium, cobalt, and manganese were relatively higher than those from nonoil-producing areas, of which the concentration of lead was significantly higher in cassava and plantain from these areas than in non-oil-exploration areas [48].

Heavy metal concentrations in food crops grown around Etelebou oil flow station in Bayelsa State had higher concentrations of iron, zinc, chromium, copper, and lead than the control values gradually accumulating over time. Of particular interest was the accumulation of lead in cassava and plantain. These findings are indicative of potential health hazards faced by the indigenous population who feed on these crops. Therefore, there is a need to closely monitor the great danger posed by the bioaccumulation of these heavy metals on the health of the population in this region [49].

## Toxicity of Heavy Metals

Heavy metals become toxic when they are not metabolized by the body and accumulate in the soft tissues [50]. Toxicity of heavy metals refers to the harmful effects that result from exposure or consumption of excessive amounts or more than the daily recommended limits. Although individual metals exhibit specific signs of toxicity, the general signs associated with cadmium, lead, arsenic, mercury, zinc, copper, and aluminium poisoning include gastrointestinal disorders, diarrhoea, stomatitis, tremor, hemoglobinuria, ataxia, paralysis, and vomiting, and convulsion, depression, and pneumonia when vapours and fumes are inhaled [51].

Ingestion or inhalation of lead can cause damage to the brain, kidneys, bone marrow, and other systems in children. Blood lead levels as low as 5µg/dL in infants and children is associated with developmental problems, such as impaired cognitive function, behavioral disorders, impaired hearing, and stunted growth [43], while levels above 75µg/dL result in coma, convulsions, and even death. Studies on pre- and postnatal cadmium exposure on intelligence quotient deficits reveal that it is a potential neurotoxicant [52]. Developmental exposure in laboratory animals indicates that operant performance and conditioned avoidance are negatively impacted as well [53]. Cadmium appears to cross the placental barrier and accumulate in the foetus, resulting in neurodegenerative disorders [54].

Nickel is an essential trace element in animals, and it is often implicated in chronic bronchitis, emphysema, impaired pulmonary function, and fibrosis [55]. Copper and chromium are important essential elements, but excessive intake causes toxicity [56]. While copper is a component of enzymes in iron metabolism whose deficiency causes anaemia [56], chromium helps to maintain blood glucose levels and is widely used in diabetes medications [57]. Toxicity of copper and chromium occur in both acute and chronic forms when taken in excess [58,59,60]. Acute copper toxicity manifests as nausea, vomiting, jaundice, and liver necrosis, damage to the proximal tubules of the kidney, and anaemia [58,59,60]. Wilson’s disease in man is a form of chronic copper toxicity that presents as mental alterations, motor abnormalities, dysphagia, ataxia, haemolytic anaemia, renal dysfunction, kidney stones, and hepatic failure [58]. Normally, chromium toxicity is due to physical contact with contaminated dust or soil, resulting in allergic dermatitis characterized by eczema [59].

## The Challenge

A number of serious health problems can result from excessive exposure to heavy metals, while consumption of contaminated foodstuffs can seriously deplete some essential nutrients in the body, leading to decreased immunological defenses, intrauterine growth retardation, impaired psychosocial behaviors, disabilities associated with malnutrition, and a high prevalence of gastrointestinal cancer [61]. Surprisingly, there seem to be no food intake diaries in Nigeria to monitor the intake of heavy metals and their levels in blood and urine; it is therefore advocated that legislation to check human exposure to lead should be based on scientific evaluation of the available data [61]. Problems associated with the land tenure system in many cities of developing and third world countries result in hazardous places, such as road verges, banks of drainage channels, and refuse dumpsites, being converted to vegetable gardens. Setbacks and shoulders of major highways are used by farmers for cultivation and even marketing of farm produce, and emissions from traffic on these roads contain lead, cadmium, zinc, and nickel, which are present in fuel as antiknock agents. This has led to contamination of air and soils on highways and the crops cultivated or marketed in these places [62].

Accumulation of heavy metals in agricultural soil through traffic emissions may result in soil contamination and elevated heavy metal uptake by crops; this affects food quality and safety [63,64]. Food chain contamination is one of the major routes of entry into the human body for these toxic pollutants [65,66]. Cadmium in particular is a United States Environmental Protection Agency (USEPA) regulated heavy metal that is used as anticorrosion and decorative coatings on metal alloys. Cadmium enters waterways through industrial effluents and galvanised pipe breakdown. It is a nonessential metal and can become toxic by displacing zinc, resulting in kidney damage [67]. In addition, epidemiological studies have revealed that cadmium may be a contributing factor in some cancers [68].

## Conclusion and Recommendation

Contaminated food is one of the main sources of exposure to heavy metals, and an increased dietary heavy metal intake may contribute to the development of various disorders. It is therefore necessary to monitor the levels of these metals in food and in the body. Long-term accumulation of heavy metals in soils results in contamination of food crops, and studies have proven that heavy-metal-contaminated food crops, fruits, and vegetables can contain levels higher than the recommended tolerable values proposed by the European Union (EU), USEPA, FAO, and WHO. Local foodstuffs commonly available in different parts of Nigeria have also been found to contribute to the body’s burden of heavy metals, and this is of public health importance.

It is recommended that people living in highly polluted urban areas should not eat large quantities of these foods in order to avoid excessive accumulation of heavy metals in the body. Dietary intake of contaminated food crops results in long-term accumulation of heavy metals, and the detrimental impact becomes apparent only after several years of exposure. Regular monitoring of foods for these toxic heavy metals from effluents and sewage is essential to prevent their excessive buildup in the food chain. It is also advisable that farmlands be located away from traffic emissions. The tradition of marketing foodstuffs, fruits, and vegetables on highways should be discouraged with appropriate legislation.

Although well-regulated in some countries, industry is a major source of many contaminants in food. Industrial activities have the potential to generate air pollutants, wastewater effluence, and solid wastes, all of which enter the food chain and cause danger to man, animals, and plants. There is a need to closely monitor the environment and put in placea ppropriate checks and balances to preserve the health of communities within the vicinity of oil exploration areas.
